# Adults with excess weight or obesity, but not with overweight, report greater pain intensities than individuals with normal weight: a systematic review and meta-analysis

**DOI:** 10.3389/fendo.2024.1340465

**Published:** 2024-03-06

**Authors:** Miguel M. Garcia, Patricia Corrales, Miguel Á. Huerta, Maciej J. Czachorowski, Visitación López-Miranda, Gema Medina-Gómez, Enrique J. Cobos, Carlos Goicoechea, Miguel Molina-Álvarez

**Affiliations:** ^1^ Area of Pharmacology, Nutrition and Bromatology, Department of Basic Health Sciences, Universidad Rey Juan Carlos, Unidad Asociada de I+D+i al Instituto de Química Médica (IQM) CSIC-URJC, Alcorcón, Spain; ^2^ High Performance Experimental Pharmacology Research Group, Universidad Rey Juan Carlos (PHARMAKOM), Alcorcón, Spain; ^3^ Grupo Multidisciplinar de Investigación y Tratamiento del Dolor (i+DOL), Alcorcón, Spain; ^4^ Area of Biochemistry, Department of Basic Health Sciences, Universidad Rey Juan Carlos, Alcorcón, Spain; ^5^ High Performance Research Group in the Study of the Molecular Mechanisms of Glucolipotoxicity and Insulin Resistance: Implications in Obesity, Diabetes and Metabolic Syndrome, Universidad Rey Juan Carlos (LIPOBETA), Alcorcón, Spain; ^6^ Consolidated Research Group on Obesity and Type 2 Diabetes: Adipose Tissue Biology (BIOFAT), Universidad Rey Juan Carlos, Alcorcón, Spain; ^7^ Department of Pharmacology and Neurosciences Institute (Biomedical Research Center), Universidad de Granada, Granada, Spain; ^8^ Biosanitary Research Institute ibs.GRANADA, Granada, Spain; ^9^ Independent investigator, London, United Kingdom; ^10^ Teófilo Hernando Institute for Drug Discovery, Madrid, Spain

**Keywords:** obesity, overweight, pain scale, body mass index, prognosis, chronic disease, analgesia

## Abstract

**Context:**

Over 1.9 billion adult people have overweight or obesity. Considered as a chronic disease itself, obesity is associated with several comorbidities. Chronic pain affects approximately 60 million people and its connection with obesity has been displayed in several studies. However, controversial results showing both lower and higher pain thresholds in subjects with obesity compared to individuals with normal weight and the different parameters used to define such association (e.g., pain severity, frequency or duration) make it hard to draw straight forward conclusions in the matter. The objective of this article is to examine the relationship between overweight and obesity (classified with BMI as recommended by WHO) and self-perceived pain intensity in adults.

**Methods:**

A literature search was conducted following PRISMA guidelines using the databases CINAHL, Cochrane Library, EMBASE, PEDro, PubMed, Scopus and Web of Science to identify original studies that provide BMI values and their associated pain intensity assessed by self-report scales. Self-report pain scores were normalized and pooled within meta-analyses. The Cochrane’s Q test and I^2^ index were used to clarify the amount of heterogeneity; meta-regression was performed to explore the relationship between each outcome and the risk of bias.

**Results:**

Of 2194 studies, 31 eligible studies were identified and appraised, 22 of which provided data for a quantitative analysis. The results herein suggested that adults with excess weight (BMI ≥ 25.0) or obesity (BMI ≥ 30.0) but not with overweight (pre-obesity) alone (BMI 25.0–29.9), are more likely to report greater intensities of pain than individuals of normal weight (BMI 18.5–24.9). Subgroup analyses regarding the pathology of the patients showed no statistically significant differences between groups. Also, influence of age in the effect size, evaluated by meta-regression, was only observed in one of the four analyses. Furthermore, the robustness of the findings was supported by two different sensitivity analyses.

**Conclusion:**

Subjects with obesity and excess weight, but not overweight, reported greater pain intensities than individuals with normal weight. This finding encourages treatment of obesity as a component of pain management. More research is required to better understand the mechanisms of these differences and the clinical utility of the findings.

**Systematic Review Registration:**

https://doi.org/10.17605/OSF.IO/RF2G3, identifier OSF.IO/RF2G3.

## 1 Introduction

According to the World Health Organization (WHO), nearly 40% of adults suffer from overweight and 13% from obesity (1). Prevalence of obesity, which is considered a chronic disease, increased worldwide in the past 50 years, reaching pandemic levels (2, 3). Both overweight and obesity are well-known risk factors for numerous chronic diseases that are among the main causes of comorbidity and mortality in Western societies including diabetes, cardiovascular conditions, and cancer, among others (4, 5). Likewise, chronic pain affects on average 20% of the general adult population (6), and this figure is likely to further increase with the demographic ageing and increased longevity in many countries (7, 8).

Associations between obesity and pain have been previously suggested, however no clear causative relationship can be unequivocally made to date (9, 10). Firm conclusions on mechanisms are complex, as many conditions can potentially come along with both obesity and pain, which are believed to make a multifactorial relationship out of it. These may include gender, age, genetic background, past experiences, social and economic status, distribution of body fat, dietary factors such as vitamin D deficiency, and the presence of ongoing pain or other chronic disorders (11–15). It is proposed that obesity can lead to pathophysiological changes, such as increased load on joints and systemic inflammation, which may contribute to the pain experience (16). Recent studies have begun to elucidate these mechanisms, suggesting that obesity may alter pain perception (17) and exacerbate existing painful conditions (18). Obesity-related chronic pain includes widespread pain in joints and other pain types such as musculoskeletal pain (19), headaches (20), abdominal pain (21), pelvic pain (22), and neuropathic pain (23), among others.

On the other hand, original research studies often make use of different methodological approaches and review articles combine similar outcomes regardless they were obtained with distinct methodologies (e.g., uncategorized BMI and BMI tertile to sextile descriptives, body fat assessed via x-ray absorptiometry, bioelectrical impedance analysis or skinfold calipers, body fat expressed as total fat mass, body fat percentage or fat mass index, verbal *vs*. visual pain rating scales, stimulus-evoked pain, pain questionnaires, patients with ongoing pain, etc.) (11, 24–26). Moreover, the pain parameter to which the different studies refer is not identical in all cases (e.g., pain intensity, sensitivity, frequency or duration) (9, 27). Given this methodological heterogeneity, the use of simple measures (e.g., 3 categories of BMI, numerical pain rating scales) could stand as excellent tools to be used in public health and social-sanitary milieus.

In this context, unidimensional pain scale scores, such as Numeric Rating Scale (NRS), constitute one of the most reliable and valid measurement tools for self-report of pain intensity (28). Concurrently, anthropometric measurements have shown correlation with pain (22, 29). However, body mass index (BMI) remains the most common used method by healthcare providers to determine overweight or obesity. While not perfect, BMI’s widespread use can be attributed to its simplicity, cost-effectiveness, and standardization by the WHO (1, 30). Additionally, recent reviews endorsing BMI (31, 32), support its current status as the best anthropometric measure. The association between BMI and other conditions, like pain, has previously been reviewed (9, 11, 33–35).

The aim of this work was to examine the relationship between the BMI groups (normal range: 18.5-24.9 kg m-2; overweight: 25.0-29.9 kg m-2 and obesity: ≥ 30 kg m-2) and the self-reported pain intensities. This research specifically aims to investigate how different BMI categories may be associated with pain intensity experienced by individuals. Although other systematic reviews have studied the relationship between obesity and pain (e.g., back pain in children, in general population without meta-analysis or even in animal models) (36–38), to our knowledge, there has not been to date a comprehensive quantitative review relating the subjective intensity of pain assessed by NRS with the classification of weight status by BMI. The positive association between NRS and BMI will encourage the treatment of obesity as a complementary intervention to be included in the interdisciplinary approach for pain management.

## 2 Materials and methods

### 2.1 Literature search and search strategy

The systematic review and meta-analysis were conducted and reported in accordance with the Cochrane Handbook for Systematic Review of Intervention (39) and the Preferred Reporting Items for Systematic Reviews and Meta-Analyses (PRISMA) (40). The experimental protocol was pre-registered on Open Science Framework (OSF) with assigned DOI: 10.17605/OSF.IO/RF2G3.

Studies were sourced by conducting a comprehensive systematic literature search in the following electronic databases: Cumulative Index of Nursing and Allied Health Literature (CINAHL), Cochrane Library, Embase, Physiotherapy Evidence Database (PEDro), PubMed, Scopus and Web of Science. The search strategy consisted of the following search terms with no restrictions in terms of date of publication: (obesity OR overweight) AND (pain). The full search strategy for each database can be found in (**Supplementary Table S1**. Subsequently, the search results were managed in Mendeley, where duplicates were removed. The initial search was conducted on March 16, 2020. An updated search was performed and independently reviewed on March 22, 2022, to identify new publications.

### 2.2 Inclusion and exclusion criteria

The search was limited to human studies and English language. Letters to the editor, case reports and conference abstracts were excluded. Studies were eligible for inclusion if they met the following criteria: (a) the study design was observational or interventional; (b) participants were adults (age 18 and over) regardless their characteristics or pathologies; (c) for interventional studies pre-intervention data was required; (d) they provided WHO general population BMI classifications (underweight, < 18.5 kg m-2; normal range, 18.5-24.9 kg m-2; overweight, 25.0-29.9 kg m-2; obesity, ≥ 30 kg m-2) (1); (e) two or more groups of participants; (f) pain intensity assessed by self-report scales (e.g., Visual Analogue Scale, VAS; Numerical Rating Scale, NRS; Numerical Pain Rating Scale, NPRS) being the minor value “no pain” and the maximum value “pain as bad as it could be”.

### 2.3 Screening and study selection

**Figure 1:**
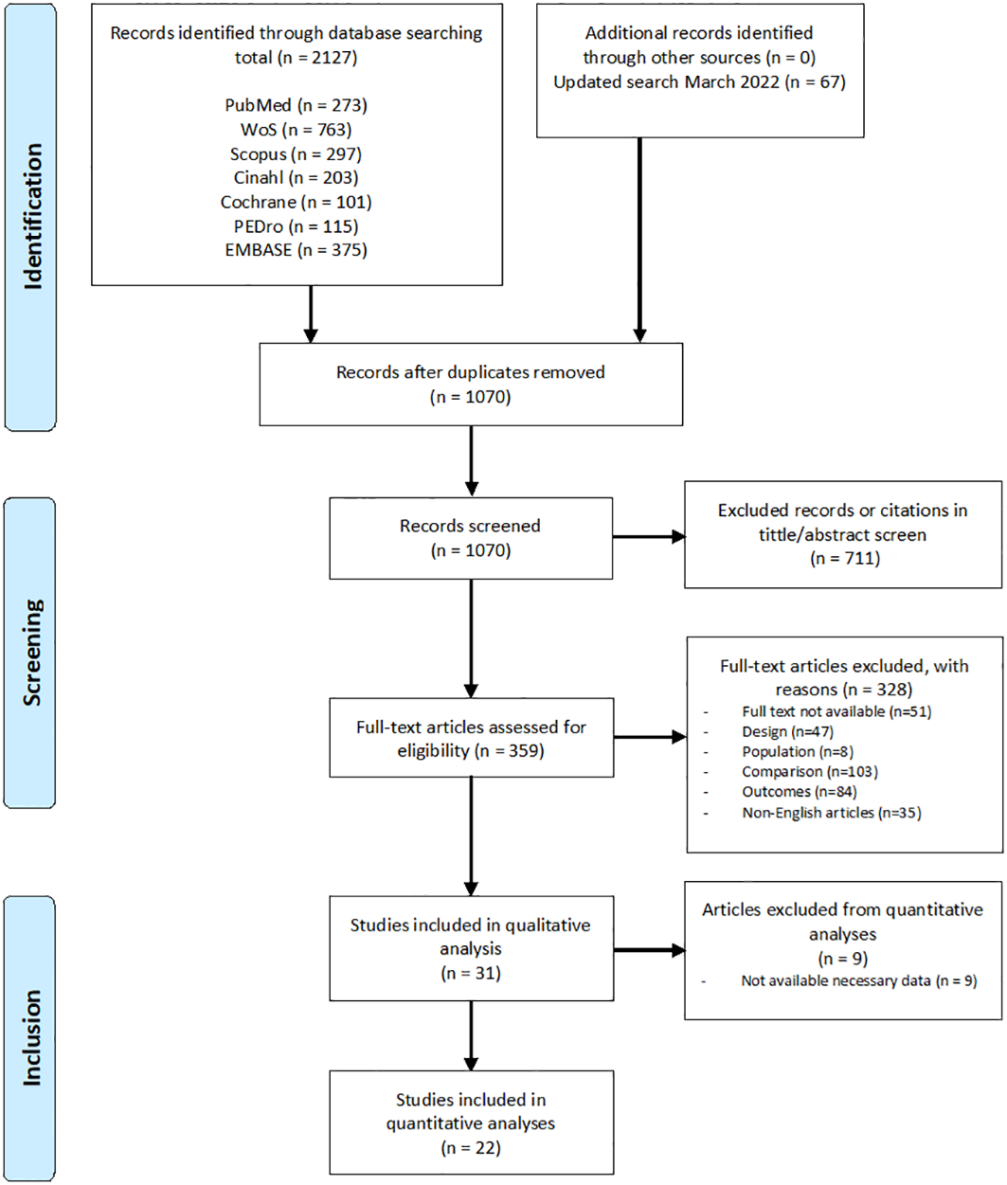
Search strategy and flow chart of the screened, excluded and analyzed studies.

The process of study selection is displayed in **Figure 1**. Screening was conducted independently by two authors (M.M.G. and P.C.). Criteria for data extraction were determined prior to initiating the review. Briefly, after duplicates were removed, titles and abstracts were screened to determine whether the citation met eligibility criteria. Subsequently, a full-text evaluation of potentially eligible studies for inclusion was performed. Disagreements were resolved by discussion between the two authors. If the authors did not reach a consensus, a third author (M.M.-A.) resolved the conflicts.

### 2.4 Data extraction

Two reviewers (M.M.G. and P.C.) independently extracted study characteristics. Authorship, year of publication, type of study, characteristics of participants (sample size, gender and age), description of the groups, pathologies, and outcome data (measurements, results and conclusions) were collected. After data compilation, discrepancies were resolved by consensus. In case of disagreement, a third reviewer (M.M.-A.) made the final decision. Any missing data was requested to the corresponding authors.

### 2.5 Risk of bias assessment

**Figure 2:**
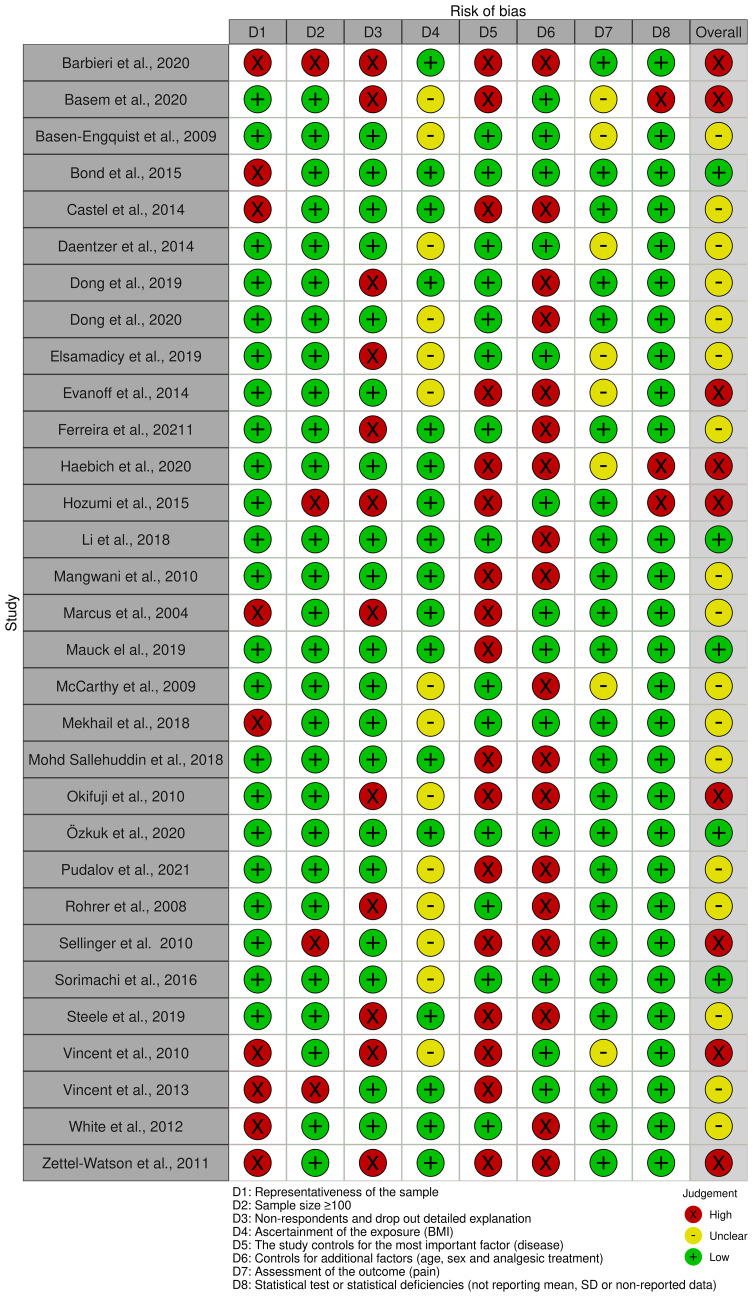
Risk of bias summary. Author’s judgments regarding each risk-of-bias item for each included study.

A modification of the Newcastle-Ottawa Scale (NOS), a standardized checklist to assess the risk of bias of nonrandomized studies in meta-analyses, was used to judge the risk of bias of the included studies. It was independently performed by two reviewers (M.A.H. and M.M.G.). The risk of bias assessment was structured into 8 main domains: (1) Representativeness of the sample: this domain evaluates whether the sample used in the study accurately reflects the population intended to be analyzed; (2) Sample size: it assesses if the study has at least a sample size over 100 participants; (3) Non-respondents and drop out detailed explanation: checks the thoroughness of reporting on participants who did not respond or dropped out during the study; (4) Ascertainment of the exposure (BMI) examines the accuracy of how the study measures the BMI; (5) control disease, the most important confounding factor: looks at how effectively the study controls for the primary confounding factor, in this case pain-related pathologies; (6) control for any additional factor: age, sex, and analgesic treatment; (7) assessment of the outcome pain: considers the precision and method of how the study measures the outcome of interest, which is pain; and (8) statistical test or statistical deficiencies: evaluates the appropriateness of the statistical tests used in the study and identifies any statistical shortcomings. A summary chart was done using *robvis*, an R package for visualizing risk-of-bias assessments (**Figure 2**) (41).

### 2.6 Data synthesis and analysis

Meta-analyses were conducted using the *metafor* and *meta* package in R, version 4.1.2 (42, 43). Since different self-report pain scales had been used in the included studies, outcome data were normalized using the standardized mean difference (SMD), which is the average difference in pain scores between two BMI groups divided by the pooled standard deviation of the two groups, computing a 95% confidence interval (CI).

For every study effect sizes (Hedges’ g statistic) were extracted from descriptive statistics. Based on Cohen’s d, Hedges’ g effect sizes were considered small (g = 0.15–0.39), medium (g = 0.40–0.74) or large (g ≥ 0.75) (44). The inverse variance statistical analysis method was used to summarize the effect sizes from the different studies subjected to the quantitative analysis. Briefly, a combined analysis (random effects) was performed, incorporating the variance in each study and between studies (39). A significance level of 0.05 was applied to determine whether differences in global effect were statistically significant between BMI groups.

Eventually, the Cochrane’s Q test (with P < 0.10 indicating asymmetry) and the Higgins-Thompson I^2^ values (null or low, 0-30%; medium, 30-50%; moderate, 50-75%; and high heterogeneity, > 75%) were used to assess the heterogeneity within the pooled studies (45).

A pathology subgroup was further defined to assess if normalized pain scores could be associated with BMI as a function of type of pathology. Additionally, a meta-regression analysis was also performed to evaluate the possible association of the results with the mean age of participants (43); and other to evaluate a possible association of the effect with the risk of bias assessed with the Newcastle-Ottawa Scale. Furthermore, two different sensitivity analyses were performed to examine the robustness of findings to decisions made during the review process. The first one was done using the leave-one-out method in order to assess the effect of a single study on the meta-analysis outcome. The second one was performed by meta-regressing the effect size and the risk of bias for each study in order to assess the influence of the risk of bias according to NOS values, using the random-effects model estimated by the method of moments (43, 46).

### 2.7 Reporting bias assessment

To assess small-study effects, we generated funnel plots for meta-analyses including at least 10 studies. Funnel plots and Egger’s test (47) were done in R using the function funnel of the *metaphor* package in order to detect publication bias. If asymmetry in the funnel plot was detected, the characteristics of the trials were reviewed to assess whether the asymmetry was likely due to publication bias or other factors such as methodological or clinical heterogeneity of the trials.

## 3 Results

### 3.1 Study selection

The search yielded 2127 citations. 67 additional articles were identified in an updated search. After duplicates removal, a total of 1070 articles were screened for suitability by title and abstract and 711 articles were excluded. Of the remaining 359 full-text articles assessed for eligibility, 328 were excluded for different reasons: 51 because the full text was not available, 47 because the design of the study was not appropriate, 8 because the population was not adequate, 103 due to issues with comparison, 84 for reasons related with the outcomes reported and 35 because they were not in English. The remaining 31 articles met the inclusion criteria and were included in the qualitative synthesis, 22 of which presented the required data for the quantitative analysis. The summary flow chart is represented in **Figure 1**.

### 3.2 Study characteristics.

The included literature spanned from 2008 (48) to 2021 (49). The studies were performed in 13 countries, mostly in the United States (n = 16). Sample size ranged from 30 (50) to 9415 (51) and 31,210 participants in sum were assessed (mean age [SD]: 53 [13.1] years; 44% were women). It should be noted that 5 studies exclusively recruited women (52–56), in one study only men were included (51), one study did not provide information on the sex (35) and in 3 studies the mean age was not provided or specified (49, 55, 57). The youngest average age was 24.1 (58) and the oldest 80.5 (59).

Among the 31 included studies, 29 were observational (15 were cross-sectional (12, 52, 53, 55, 56, 58–67), 7 retrospective (28, 35, 48, 49, 68–70), 6 prospective (51, 57, 71–74) and 1 combined analysis of retrospective and prospective occurrences (75) in cohort studies) and 2 were interventional [clinical trials (50, 54)]. BMI classification was monitored for all the included studies according to the WHO standards; however, not all the studies made use of a complete subclassification stratified in four classes. Most studies classified the participants in normal range (BMI: 18.5-24.9), overweight or pre-obesity (BMI: 25.0-29.9) and obesity (BMI ≥ 30.0) (28, 48, 49, 51, 52, 54, 56, 63–66, 72–74). However, 10 articles (35, 51, 52, 58–60, 62, 63, 68, 70) recruited underweight patients, of which 3 studies (52, 63, 70) added such data to the normal range category, and two (51, 68) left them out of their quantitative analysis. Although a subclassification for obesity was carried out in 11 studies (12, 49, 55, 57, 59, 60, 65–68, 70), none included the same subgroups in their studies.

Regarding the pathologies examined, chronic pain conditions were predominant (12, 28, 35, 48, 53, 54, 57, 60, 61, 63–70, 72–75), with back pain (12, 28, 57, 65, 67, 69, 70, 72–75) being the most prevalent pathology in the included studies. Some studies included general population without specifying the existence of any underlying disease among participants (50, 51, 55, 56, 59, 62).

Finally, subjective pain intensities were assessed as the means of different pain-rating scales: VAS (28, 53, 55, 56, 58, 63, 64, 66, 72, 74) and NRS (12, 35, 48–54, 57, 59–63, 65, 67–71, 73, 75, 76) (alternatively named NPRS). The main selected descriptive characteristics of the studies are summarized in **Table 1**.

**Table 1:** Descriptive characteristics of the included studies.

Reference, publication year [country]	Study type	Sample size	Mean age	% Female	BMI classification (%)	Participants’ condition	Pain scale
Barbieri et al. (29), 2020 [Brazil]	Clinical trial	30	30.8	56.7	18.5-24.9 (50.0) 25.0-34.9 (50.0)	Not specified (general population)	NRS
Basem et al. (36), 2020 [USA]	Observational, restrospective	2509	59.0	not provided	<18.5 (2.7) 25.0-29.9 (68.0) ≥30.0 (29.3)	All chronic pains	NRS
Basen-Engquist et al. (31), 2009 [USA]	Cross-sectional	112	59.9	100.0	<25.0 (33.9) 25.0-29.9 (15.2) ≥30.0 (50.9)	Endometrial cancer	NRS
Bond et al. (32), 2015 [USA]	Cross-sectional	105	38.1	100.0	25.0-29.9 (not specified) ≥30.0 (not specified)	Migraine	VAS
Castel et al. (33), 2014 [Spain]	Clinical trial	130	49.2	100.0	18.5-24.9 (31.5) 25.0-29.9 (37.7) ≥30.0 (30.8)	Fibromyalgia	NRS
Daentzer et al. (15), 2015 [Germany]	Observational, restrospective	128	60.9	58.6	<25.0 (35.2) 25.0-29.9 (36.7) ≥30.0 (28.1)	Low back pain	VAS
Dong et al. (48), 2019 [Sweden]	Observational, retrospective	872	45.8	80.3	<18.5 (1.4) 18.5-24.9 (32.1) 25.0-29.9 (40.8) 30.0-34.9 (18.5) ≥35.0 (7.2)	Non-malignant chronic pain	NRS
Dong et al. (40), 2020 [Sweden]	Cross-sectional	3110	44.5	74.8	<18.5 (1.5) 18.5-24.9 (37.5) 25.0-29.9 (35.8) 30.0-34.5 (17.4) ≥35.0 (7.8)	Non-malignant chronic pain	NRS
Elsamadicy et al. (49), 2019 [USA]	Observational, restrospective	112	52.4	72.3	<30.0 (70.5) ≥30.0 (29.5)	Spine deformity	NRS
Evanoff et al. (30), 2014 [France]	Observational, prospective	9415	68.0	0.0	<25.0 (inconsistent) 25.0-29.9 (inconsistent) ≥30.0 (inconsistent)	Not specified (general population)	NRS (1–8)
Ferreira et al. (38), 2021 [Brazil]	Cross-sectional	100	24.1	60.0	<18.5 (0.0) 18.5-24.9 (62.0) 25.0-29.9 (24.0) ≥30.0 (14.0)	Knee pain	VAS
Haebich et al. (51), 2020 [Australia]	Observational, prospective	191	68.0	56.5	<30.0 (59.2) ≥30.0 (40.8)	Hip pain	NRS
Hozumi et al. (41), 2016 [Japan]	Cross-sectional	44	60.0	32.7	18.5-24.9 (68.2) ≥25.0 (31.8)	Neuropathic pain	NRS
Li et al. (42), 2018 [China]	Cross-sectional	6524	71.1	56.5	<18.5 (4.6) 18.5-23.9 (50.1) 24.0-29.9 (36.0) ≥30.0 (9.3)	Not specified (general population ≥60)	NRS
Mangwani et al. (52), 2010 [UK]	Observational, prospective	140	38.0	37.9	<25.0 (47.9) 25.0-29.9 (31.4) ≥30.0 (20.7)	Low back pain	VAS
Marcus et al. (53), 2004 [USA]	Observational, prospective	372	46.4	63.4	<25.0 (36.8) 25.0-30.0 (27.2) ≥30.0 (36.0)	Mixed chronic pain	NRS
Mauck et al. (37), 2019 [USA]	Observational, prospective	963	Not specified (18–65)	57.4	18.5-24.9 (40.2) 25.0-29.9 (30.7) 30.0-34.9 (16.8) ≥35.0 (12.3)	Motor vehicle collision	NRS
McCarthy et al. (39), 2009 [USA]	Cross-sectional	840	80.5	62.4	<18.5 18.5-24.9 25.0-29.9 30.0-34.9 ≥35.0	Not specified (general population ≥70)	NRS
Mekhail et al. (50), 2018 [USA]	Observational, restrospective	181	55.0	55.8	<25.0 (18.2) 25.0-29.9 (39.8) 30.0-39.9 (34.8) ≥40.0 (7.2)	Chronic spine-related conditions	NRS
Mohd Sallehuddin et al. (34), 2018 [Malaysia]	Cross-sectional	156	not specified (18–59)	100.0	25.0-29.9 (50) 30.0-35.0 (30.8) ≥35.0 (19.2)	Not specified (housewives)	VAS
Okifuji et al. (43), 2010 [USA]	Cross-sectional	215	45.3	94.9	<18.5 (1.9) 18.5-24.9 (21.8) 25.0-29.9 (29.8) ≥30.0 (46.5)	Fibromyalgia	VAS NRS
Özkuk et al. (44), 2020 [Turkey]	Cross-sectional	191	58.7	75.4	18.5-24.9 (20.4) 25.0-29.9 (37.2) ≥30.0 (42.4)	Chronic shoulder pain	VAS
Pudalov et al. (27), 2021 [USA]	Observational, restrospective	714	47.6	68.8	18.5-24.9 (22.5) 25.0-29.9 (27.3) ≥30.0 (50.2)	Chronic non-malignant pain	NRS
Rohrer et al. (28), 2008 [USA]	Observational, restrospective	577	not provided	63.2	<25.0 (30.3) 25.0-29.9 (31.4) 30.0-34.5 (21.8) ≥35.0 (16.5)	Not specified (clinical center)	NRS
Sellinger et al. (55), 2010 [USA]	Observational, combined restrospective and prospective	74	58.2	10.8	<30.0 (43.2) >30.0 (56.8)	Chronic low back pain (Veterans)	NRS
Sorimachi et al. (54), 2016 [Finland]	Observational, prospective	805	61.0	67.6	<25.0 (25.4) 25.0-29.9 (45.3) ≥30.0 (29.3)	Spinal fusion surgery	VAS
Steele et al. (35), 2019 [Australia]	Cross-sectional	378	45.6	100.0	18.5-24.9 (43.1) 25.0-29.9 (27.3) ≥30.0 (29.6)	Not specified (general population)	VAS
Vincent et al. (45), 2010 [USA]	Cross-sectional	278	37.4	43.5	<25.0 (27.0) 25.0-29.9 (33.8) 30.0-39.9 (26.3) ≥40.0 (12.9)	Knee pain	NRS
Vincent et al. (10), 2013 [USA]	Cross-sectional	55	67.8	65.5	25.0-29.9 (30.9) 30.0-34.9 (47.3) ≥35.0 (21.8)	Chronic low back pain	NRS
White et al. (46), 2012 [USA]	Cross-sectional	1788	67.2	60.0	<25.0 (15.0) 25.0-29.9 (36.0) 30.0-34.9 (29.0) ≥35.0 (20.0)	Knee osteoarthritis	VAS
Zettel-Watson et al. (47), 2011 [USA]	Cross-sectional	101	52.1	80.2	25.0-29.5 (28.7) 30.0-39.5 (53.5) ≥40.0 (17.8)	Chronic pain (hispanic)	NRS

BMI, body mass index; NRS, numerical rating scale; VAS, visual analogue scale.

### 3.3 Risk of bias

Among 31 eligible trials, 5 were considered to be articles with low risk of bias (only 1 or 2 domains classified as high risk of bias) (53, 57, 62, 64, 74), 17 were considered to have moderate risk of bias (3 or 4 domains classified as high risk of bias) (12, 28, 48, 49, 52, 54–56, 58–60, 66, 68–70, 72, 73) and 9 were classified as articles with a high risk of bias (more than 4 domains classified as high risk of bias) (35, 50, 51, 61, 63, 65, 67, 71, 75) (**Figure 2**). The influence of the risk of bias in the effect observed was evaluated for each forest plot doing a sensitivity analysis (view Meta-analyses section).

### 3.4 Meta-analyses

Due to insufficient reporting on raw data or due to inadaptable reporting of results, 9 of the studies (12, 35, 49–53, 57, 59) that were reviewed were found to be incompatible for the meta-analysis and therefore they were redacted from our study. The results of the remaining 22 studies (28, 48, 54–56, 58, 60–75) were pooled into different groups according to body mass indexes – normal weight for 18.5-24.9, excess weight (overweight plus obesity) for ≥ 25.0, overweight for 25.0-29.9 and obesity for ≥ 30.0; subsequently pain intensities were analyzed between groups. The analysis results were displayed in forest plots for a comprehensive reading of the studies. All data included in each meta-analysis were split into subgroups according to the conditions of the participants (their pathologies) and new meta-analyses were performed to infer their contribution to pain intensity in the differences observed between BMI groups. Additionally, to test the hypothesis that age could exert an influence in the given results, meta-regressions were performed (43, 46).

#### 3.4.1 Comparison of self-report pain intensity in people of normal weight versus people with excess weight (overweight or pre-obesity plus obesity)

**Figure 3:**
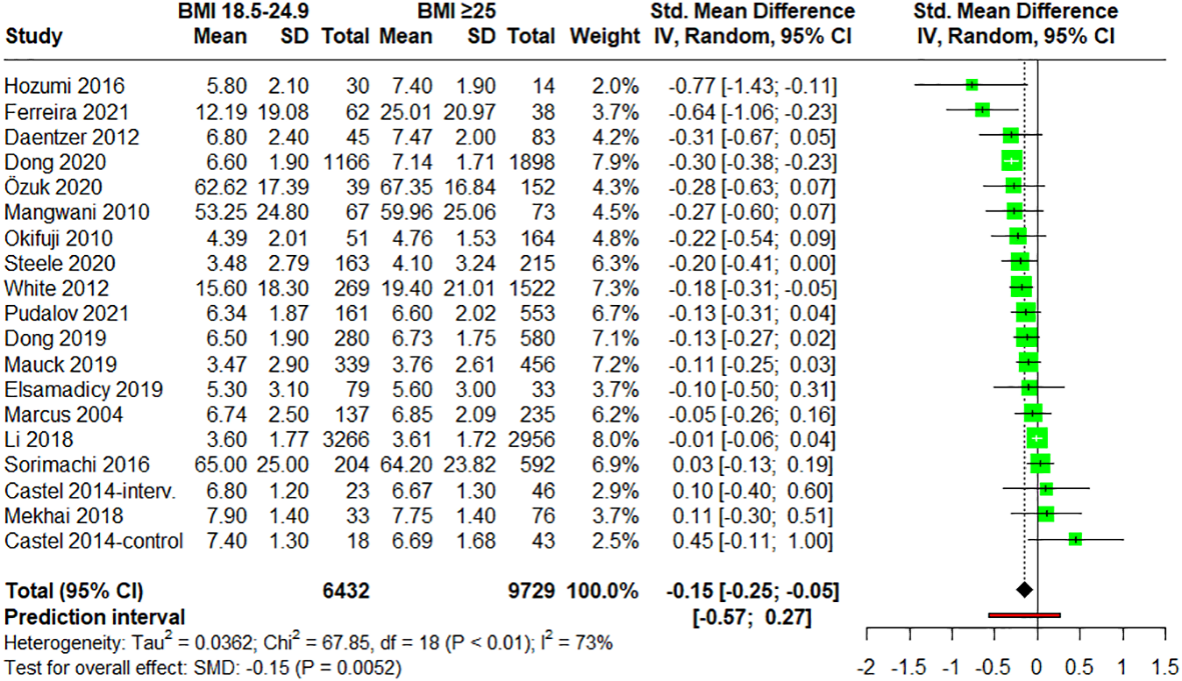
Forest plot of meta-analysis for studies assessing pain intensity in adults with normal weight (BMI = 18.5-24.9) versus adults with excess weight (overweight and obesity) (BMI ≥ 25.0). Negative values indicate that pain intensities in adults of normal weight are lower than those for adults with excess weight (overweight and obesity). SD, standard deviation; CI, confidence interval.

First, the relationship between pain measures in patients with normal weight (BMI = 18.5-24.9) *vs*. patients with overweight plus obesity (BMI ≥ 25) was studied. The meta-analysis included 17 studies (28, 48, 54, 56–58, 60, 62–66, 68, 69, 72–74) that provided data for groups with normal weight and for groups with BMI over 25; and showed that people with BMI over 25 reported higher pain intensities (statistically significant differences). The overall effect of weight status on self-reported pain intensity was small (SMD = –0.15; 95% CI = –0.25 to –0.05; P = 0.0052; N = 16,161; n = 19 trials) and moderate heterogeneity (I^2^ = 73.5%; QE = 67.85, P < 0.0001) was found (**Figure 3**). The subgroup analysis regarding the pathology of the included patients (fibromyalgia, low back pain, chronic pain, back pain, knee pain, neuropathic pain, shoulder pain or general population) showed no significant differences (P = 0.2551), so apparently the effect was not influenced by this variable (**Supplementary Figure S1**). Moreover, no significant differences (P = 0.2065) were observed in the meta-regression between mean ages of the patients and the effect size (**Supplementary Figure S2**).

#### 3.4.2 Comparison of self-report pain intensity in people of normal weight versus people with overweight

**Figure 4:**
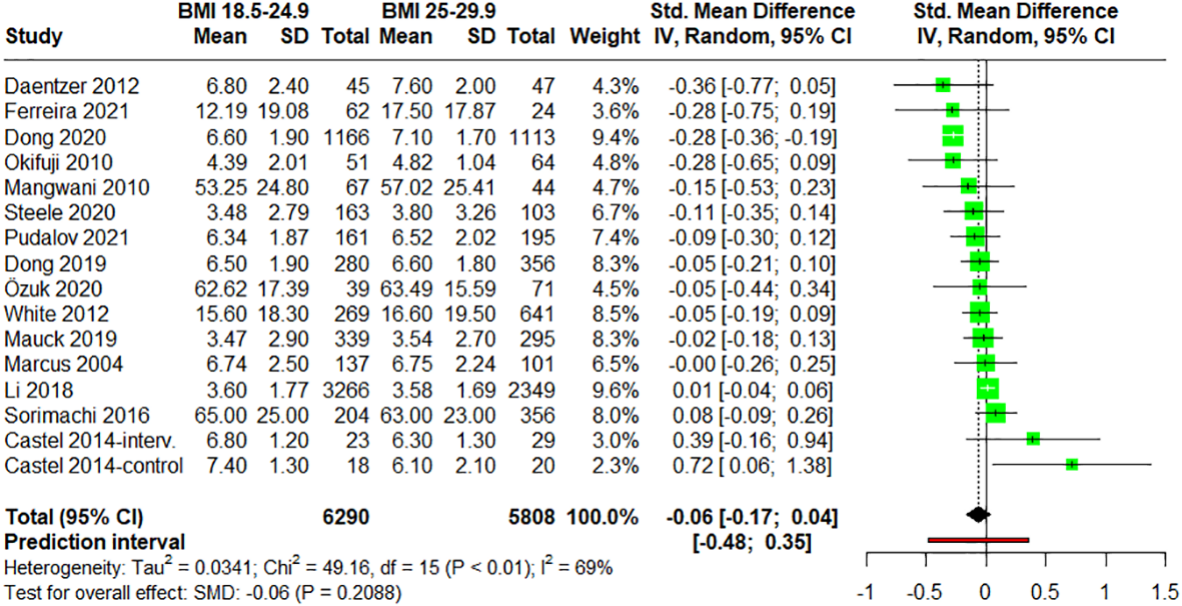
Forest plot of meta-analysis for studies assessing pain intensity in adults with normal weight (BMI = 18.5-24.9) versus adults with overweight (BMI = 25-29.9). Negative values indicate that pain intensities in adults of normal weight are lower than those for adults with overweight (pre-obesity). SD, standard deviation; CI, confidence interval.

After studying the relationship between pain measures and people with normal weight *vs*. people with overweight plus obesity, participants of normal range (BMI = 18.5-24.9) were compared against participants with overweight (pre-obesity) (BMI = 25-29.9). 15 studies (12, 28, 48, 54, 56, 58, 60, 62–64, 66, 68, 72–74) provided data on participants with BMI between 25.0 and 29.9 (**Figure 4**). When the data were meta-analyzed, the results did not show any statistically significant difference between groups (SMD = –0.06; 95% CI = –0.17 to 0.04; P = 0.2088; N = 12,098; n = 16 trials), suggesting that pain scores were comparable between the two groups. The heterogeneity was found to be moderate (I^2^ = 69.5%; QE = 49.16, P < 0.0001) (**Figure 4**). In terms of the pathology, the subgroup analysis showed no statistically significant differences between pathologies (P = 0.57) (**Supplementary Figure S3**). Additionally, neither significant differences were observed in the meta-regression analysis between the mean age of the patients and the effect size (P = 0.5248) (**Supplementary Figure S4**).

#### 3.4.3 Comparison of self-reported pain intensity in people of normal weight versus people with obesity

**Figure 5:**
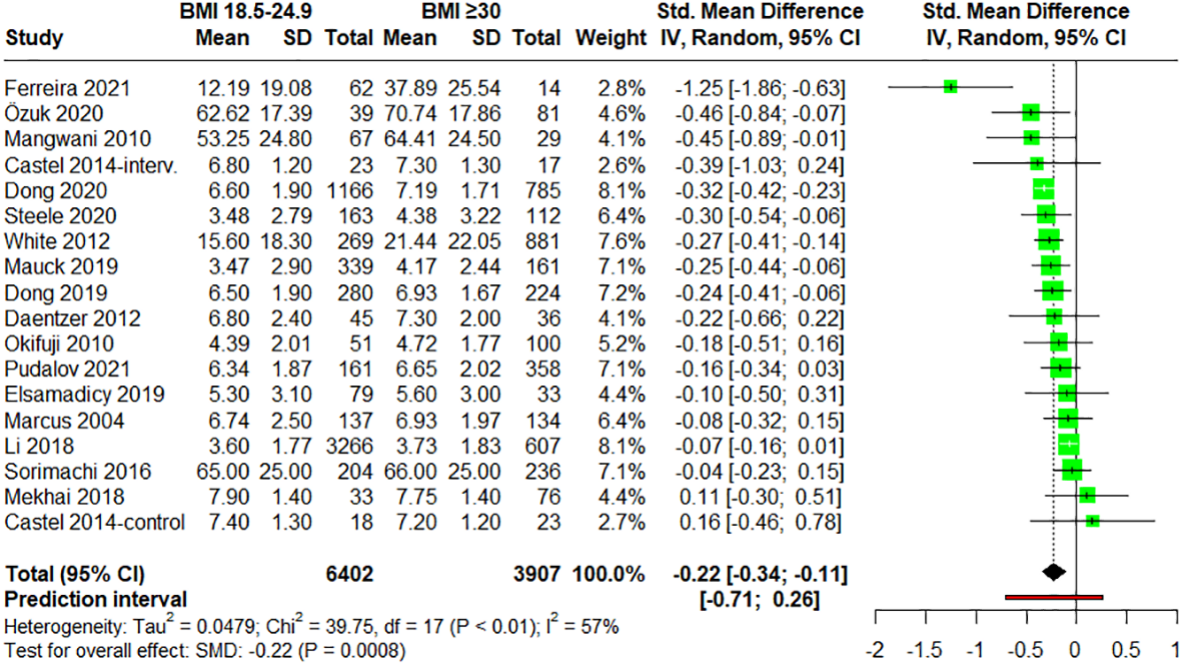
Forest plot of meta-analysis for studies assessing pain intensity in adults with normal weight (BMI = 18.5-24.9) versus adults with obesity (BMI ≥30). Negative values indicate that pain intensities in adults of normal weight are lower than those for adults with obesity. SD, standard deviation; CI, confidence interval.

17 studies (28, 48, 54, 56, 58, 60, 62–66, 68–70, 72, 73, 75) provided data for groups with BMI over 30. The forest plot for the meta-analysis comparing pain intensities in normal weight (BMI = 18.5-24.9) patients *vs*. obesity (BMI ≥ 30) patients is showed in **Figure 5**. Statistically significant differences were observed between these two groups suggesting that adults with obesity reported higher pain intensities (small effect size) (SMD = –0.22; 95% CI = –0.34 to –0.11; P = 0.0008; N = 10,309; n = 18 comparisons). Again, moderate heterogeneity was found (I^2^ = 57.2%; QE = 39.75, P = 0.0014). When evaluating the influence of the pathologies on pain perception with a subgroup analysis, no statistically significant differences were found between groups (P = 0.2300) (**Supplementary Figure S5**). Additionally, statistical analysis reveals a significant negative intercept (-1.0363, P = 0.0012) and a small but significant positive effect of age (0.0127 per year, P = 0.0293) on pain, underscoring the nuanced yet significant influence of age on pain within our regression model. (**Supplementary Figure S6**).

#### 3.4.4 Comparison of self-report pain intensity in people with overweight versus people with obesity

**Figure 6:**
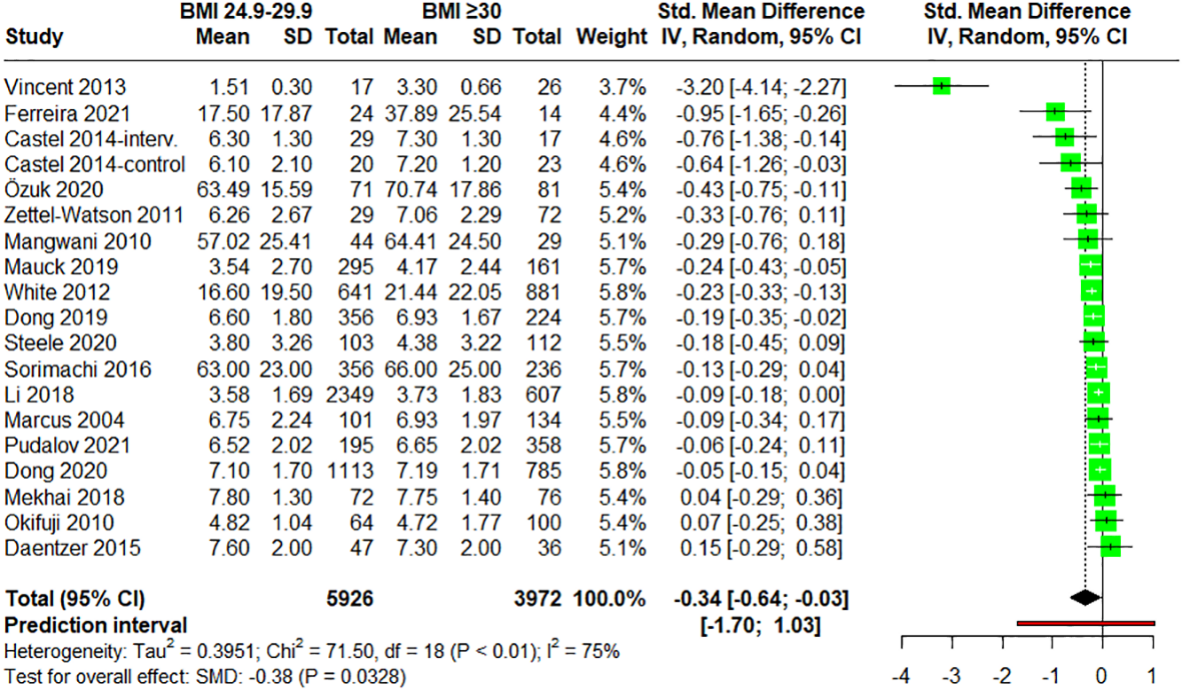
Forest plot of meta-analysis for studies assessing pain intensity in adults with overweight (BMI = 25-29.9) versus adults with obesity (BMI ≥30). Negative values indicate that pain intensities in adults with overweight are lower than those for adults with obesity. SD, standard deviation; CI, confidence interval.

Finally, when analyzing normalized pain intensities of participants with overweight (BMI = 25-29.9) *vs*. participants with obesity (BMI ≥ 30) (n = 18) (28, 48, 54–56, 58, 60, 62–68, 70, 72–74), statistically significant differences were found with a small effect size (SMD = –0.34; 95% CI = –0.64 to –0.03; P = 0.0328; N = 9,898; n = 19 trials), indicating lower pain scores in overweight participants. Heterogeneity was however moderate (I^2^ = 74.8%; QE = 71.50, P < 0.0001) (**Figure 6**). When subgroup analyses regarding the pathology of the patients were performed, no statistically significant differences were found between groups (P = 0.3370) (**Supplementary Figure S7**). Furthermore, no significant differences were observed in the meta-regression between the mean age of the patients and the effect size (P = 0.3412) (**Supplementary Figure S8**).

### 3.5 Sensitivity analysis

Sensitivity analysis with leave-one-out method for the first meta-analysis (normal weight *vs*. people with excess weight) showed that excluding individual studies had no relevant influence on the results (**Supplementary Figure S9**). For the second meta-analysis (normal weight versus people with overweight) no relevant influence on the results was found, given that no study was determinant in explaining the absence of significant differences between groups (**Supplementary Figure S10**). When people with normal weight *vs*. people with obesity were compared, similar results were found (**Supplementary Figure S11**). Finally, in overweight *vs*. obesity comparison, Vincent et al., 2013 (12) was identified to cause an overestimation of the effect size. Leaving out the study, the effect size was reduced, nevertheless, the meta-analysis continued presenting statistical differences (SMD = –0.18; 95% CI = –0.29 to –0.07) (**Supplementary Figure S12**).

Another sensitivity analysis was performed by meta-regressing the NOS scores (view 3.3. Risk of bias) and the standardized mean difference values in order to observe a possible differential contribution of the articles with high a risk of bias. Risk of bias did not show influence in the effect size for any comparison: normal *vs*. overweight and obesity (P = 0.4094); normal *vs*. overweight (P = 0.8685); normal *vs*. obesity (P = 0.8769); overweight *vs*. obesity (P = 0.9129) (**Supplementary Figures S13**–**S16**, respectively). These analyses support the stability of the meta-analysis and the robustness of the results.

### 3.6 Publication bias assessment

Inspection of the funnel plots for the meta-analyses indicated no clear risk of publication bias for most comparisons, as the standard errors by the effect size estimates showed a symmetrical distribution (**Supplementary Figures S17**–**S20**). However, the funnel plot for the comparison between overweight *vs*. obese was asymmetrical (**Supplementary Figure S21**), suggesting potential publication bias or other small-study effects. Caution is warranted when interpreting these particular results.

## 4 Discussion

### 4.1 Main findings

In this systematic review and meta-analysis of 31,210 participants from diverse international cohorts, people with excess weight (overweight or pre-obesity plus obesity; BMI ≥ 25.0) or obesity reported higher pain intensities than those with normal weight (BMI = 18.5-24.9). Also, individuals with obesity reported higher pain intensities than the overweight individuals. However, overweight (pre-obesity, BMI = 25.0-29.9) patients did not present higher pain intensities than those with normal weight. For this reason, this systematic review and meta-analysis provide quantitative evidence that BMI greater than or equal to 30.0 is associated with greater self-reported pain intensity in the general adult population. Some pathophysiological changes that occur in patients with obesity can explain this increase in the pain intensity perceived. The proinflammatory state of obesity patients [e.g., higher levels of IL-6, TNF, prostaglandins, and others (77)] may contribute to increase pain intensity (9, 27, 63). Also, the mechanical stress associated to the high weight could overload joints and muscles causing injury (27, 78). This generates pain due to the activation of mechanoreceptors on chondrocytes and an increase in metalloproteases and interleukin 1 (IL-1), which also contribute to the increased proinflammatory status (9).

Additionally, the increased pain perception associated with obesity or overweight plus obesity was not related to specific pathologies as no statistically significant differences were found in the subgroup analysis regarding pathologies (**Supplementary Figure S1**, **S5**, **S7**). Neither pathology influence was found between normal weight and overweight regarding pain perception (**Supplementary Figure S3**). However, the observed absence of differences between specific pain-related pathologies may be illusory and related with the low representation of some subgroups (the majority included 5 or less evaluations). In fact, the analyses reflected some tendencies (e.g., pain intensities of chronic pain patients were apparently greater in the higher BMI groups than in their normal-weighted counterparts, while fibromyalgia and back pain showed a contrary pattern). In addition, several subgroup analyses were near statistical significance. For all the above, in a bigger analysis, it would be expected to find differences between pathologies, which is reasonable, as each pain type has different pathophysiological particularities that could be differently modulated by BMI categories (79–81), also pain intensities can differ between pain-related pathologies (82–84). Similar results were found when analyzing the association between the variable mean age and the effect size by meta-regression. In this regard, dependence between these two variables was only found in the meta-regression associated to the third meta-analysis (**Supplementary Figure S6**), but not in any of the other three analyses performed (**Supplementary Figure S2**, **S4**, **S8**). Again, the absence of influence could be related with the sample size as the interrelationship between pain and age is clearly stablished (84, 85). Also, high BMI (mainly obesity) is known to have more impact on quality of life (burden of disease) as age increases (86, 87). So, it would be plausible that the positive association of BMI with pain intensity was potentiated by the age.

In some way, the results herein are consistent with previous prevalence studies that concluded that people with obesity, defined according to their BMI, were in effect more prone to suffering from daily pain (88, 89). Similarly, a previous study reported the association of obesity with different pain conditions (e.g., low back pain, migraine headache, fibromyalgia, abdominal and kidney pain) (90). However, although not necessarily mutually exclusive, our results would differ from other studies that indicate that chronic pain would be more frequent among patients with overweight status (BMI: 25.0-29.9) than in patients with normal weight range (91). Although previous reviews have also tried to address the topic (with similar conclusion), most focus on the prevalence or incidence of pain (91–93), and others did not perform quantitative analysis (36, 37). Directives are clear, but specific links between pain and obesity remain frequently vague and imprecise (94). This may be justified, as commented previously, by the complex interrelationships among pain, body weight and the intrinsic conditions of the patients, but also by the large diversity of study designs (37, 95). In sum, the data herein support the idea that people with excess weight or obesity report higher pain scores than the normal-weighted population.

### 4.2 Strengths and limitations

The results of this systematic review and meta-analysis should be considered according to its potential strengths and limitations. Potential strengths include its preregistered design, comprehensive search strategy, updated systematic study inclusion, quantitative evidence and use of formal tests for heterogeneity (Q-statistic; I^2^). Additionally, seven different databases were consulted, a large number of participants were gathered (31,210 individuals overall) and the different country origin of the articles may allow to extend the results herein globally. Not less important, only NRS and BMI were used so that the outcomes herein were based on an identical methodological approach. In fact, to our knowledge, this is the first systematic review and meta-analysis analyzing the correlation between NRS and BMI in adult population. However, there are also limitations to our review. First, our study did not contemplate underweight patients and, in this regard, qualitative associations between physical pain and malnutrition have previously been suggested (96). Second, the WHO stratifies patients into different categories according to their BMI, but this index does not strictly reflect the adiposity nor can distinguish two individuals with similar BMI and different body composition. That is, making use of BMI to indicate weight status may misclassify some people with excessive muscularity (97, 98). Given the scarce number of articles making use of all same common parameters including waist circumference, body fat analysis and BMI (97, 99), we were not able to implement a different assessment for obesity. Third, sex differences are also significant obesity-related metabolic risk factors, and they seem to play a predictive role in certain pain-associated complications (89). These differences in body composition and adipose mass distribution could contribute to sex-dimorphic obesity and its association with different painful conditions. When pain is the main outcome, sex can be an important bias and analyzing the data separately could be relevant (100). A gender-based comparison could not be performed given that the articles did not report data separately for male and female study participants, except for one study that exclusively included men (97) and five that only recruited women (52–56). Fourth, regardless of the results presented herein, we are aware that the relationship between obesity and pain is not simple but chronic inflammation, the localization of fat mass (related to sexual dimorphism), genetic and environmental factors may be significant contributors to the association between obesity and pain. Finally, although this work provides aggregate and recent data on all the literature that we could gathered from the bibliographic databases mentioned above, heterogeneity (I^2^) amongst studies was moderate, with minimal values of 57.2% and maximal of 74.8%, and effect sizes were relatively small in all outcomes (g = –0.15, –0.25 and –0.05 for normal weight *vs*. excess weight, normal weight *vs*. obesity and overweight *vs*. obesity, respectively). These effect sizes may serve however for future comparisons with upcoming reviews.

### 4.3 Clinical relevance of the results

This work shows that BMI higher than 30 (obesity) is clearly associated with higher pain intensities. Previous evidence shows that patients with obesity suffering pain may experience a reduction in pain intensity after weight loss or obesity treatment (101–104). Accordingly, pain clinicians should pay attention to BMI and consider deriving the patient to the endocrinology service for an adequate management of obesity, which may improve the results of the pain management intervention. In this sense, treatment for obesity could constitute one more element that could be added to the interdisciplinary pain management, which is usually the best strategy in the treatment of pain (105, 106).

## 5 Conclusion

To the best of our knowledge, this is the first systematic review and meta-analysis to examine the association of self-rating pain scores and body mass indexes in general adult population. The results indicated that adults with excess weight (BMI ≥ 25.0) or obesity (BMI ≥ 30.0) but not with overweight (pre-obesity) alone (BMI 25.0–29.9), are more likely to report greater intensities of pain than individuals of normal weight (BMI 18.5–24.9). These findings encourage the treatment of obesity and the control of body mass index (weight loss) as key complementary interventions for pain management.

## Data availability statement

The original contributions presented in the study are included in the article/**Supplementary Material**. Further inquiries can be directed to the corresponding author.

## Author contributions

MG: Conceptualization, Data curation, Investigation, Methodology, Project administration, Resources, Writing – original draft, Writing – review & editing. PC: Conceptualization, Data curation, Investigation, Methodology, Resources, Writing – original draft. MH: Investigation, Methodology, Writing – original draft, Writing – review & editing. MC: Validation, Writing – review & editing. VL-M: Supervision, Validation, Writing – review & editing. GM-G: Supervision, Writing – review & editing. EC: Supervision, Writing – review & editing. CG: Supervision, Writing – review & editing. MM-A: Conceptualization, Data curation, Methodology, Resources, Validation, Writing – original draft.
