# Supplementary material for: Adults with excess weight or obesity, but not with overweight, report greater pain intensities than individuals with normal weight: a systematic review and meta-analysis
Source: Front Endocrinol (Lausanne). 2024 Mar 6;15:1340465. doi: 10.3389/fendo.2024.1340465 (PMC10950917; doi:10.3389/fendo.2024.1340465)
Supplement: Supplementary file 1 [file DataSheet_1.docx]

**Supplementary Material**

**Table S1. Search strategies for all included search engines.**

| CINAHL (EBSCO) | |
| --- | --- |
| 1. | TI (obesity OR overweight) |
| 2. | TI pain |
| 3. | #1 AND #2 |
| The Cochrane Library | |
| 1. | (obesity):ti OR (overweight):ti |
| 2. | (pain):ti |
| 3. | #1 AND #2 |
| EMBASE (ELSEVIER) | |
| 1. | ‘overweight:ti’ OR ‘obesity:ti’ |
| 2. | ‘pain:ti’ |
| 3. | #1 AND #2 |
| PEDro | |
| 1. | obesity |
| 2. | overweight |
| 3. | pain |
| 4. | #1 AND #3 |
| 5. | #2 AND #3 |
| PubMed (NLM) | |
| 1. | obesity[Title] OR overweight[Title] |
| 2. | pain[Title] |
| 3. | #1 AND #2 |
| Scopus (ELSEVIER) | |
| 1. | TITLE (obesity OR overweight) |
| 2. | TITLE (pain) |
| 3. | #1 AND #2 |
| Web of Science (ELSEVIER) | |
| 1. | obesity (Title) OR overweight (Title) |
| 2. | pain (Title) |
| 3. | #1 AND #2 |


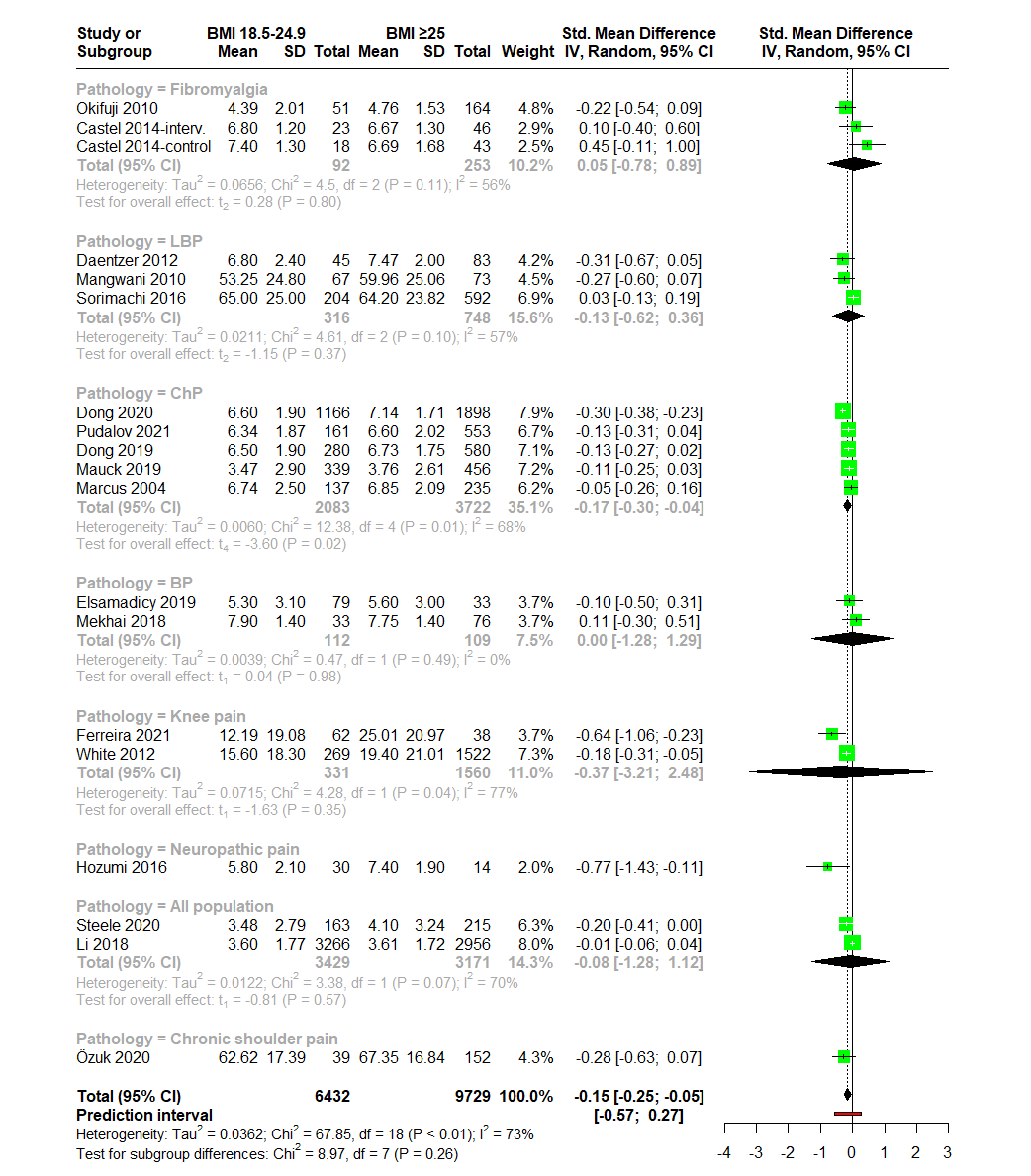


**Figure S1. Forest plot of subgroup analysis regarding pathology for studies assessing pain intensity in adults with normal weight (BMI = 18.5-24.9) *versus* adults with excess weight** **(overweight and obesity) (BMI ≥ 25).** Negative values indicate that pain intensities in adults of normal weight are lower than those for adults with excess weight. SD: standard deviation; CI: confidence interval; LBP: low back pain; ChP: chronic pain; BP: back pain.


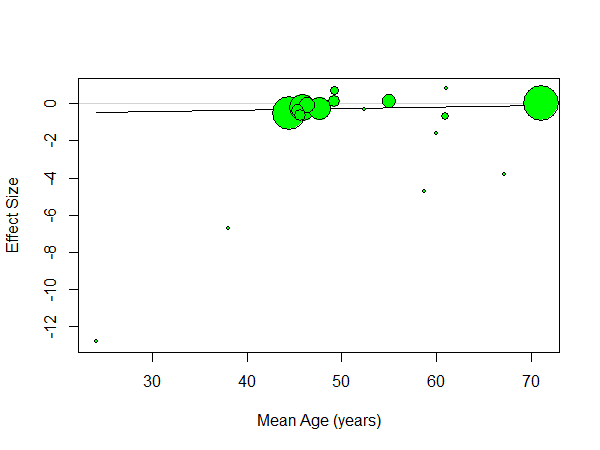


**Figure S2. Bubble plot (meta-regression) of age influence on between-group effect size for studies assessing pain intensity in adults with** **normal weight (BMI = 18.5-24.9) *versus* adults with excess weight (overweight and obesity) (BMI ≥ 25).**


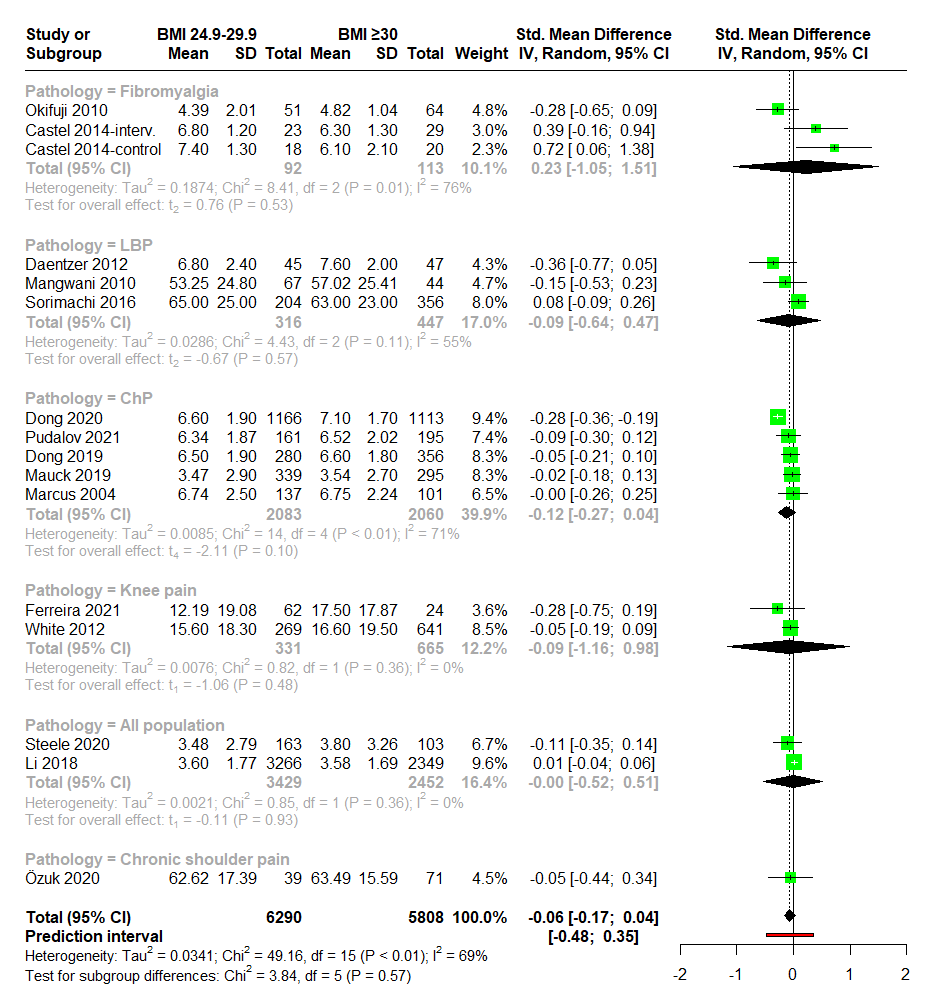


**Figure S3. Forest plot of subgroup analysis regarding pathology for studies assessing pain intensity in adults with normal weight (BMI = 18.5-24.9) *versus* adults with overweight (BMI = 25-29.9).** Negative values indicate that pain intensities in adults of normal weight are lower than those for adults with overweight. SD: standard deviation; CI: confidence interval; LBP: low back pain; ChP: chronic pain; BP: back pain.


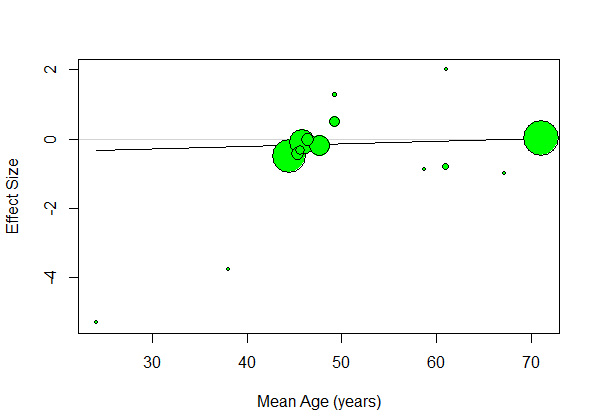


**Figure S4. Bubble plot (meta-regression) of age influence on between-group effect size for studies assessing pain intensity in adults with normal weight (BMI = 18.5-24.9) *versus* adults with overweight (BMI = 25-29.9).**


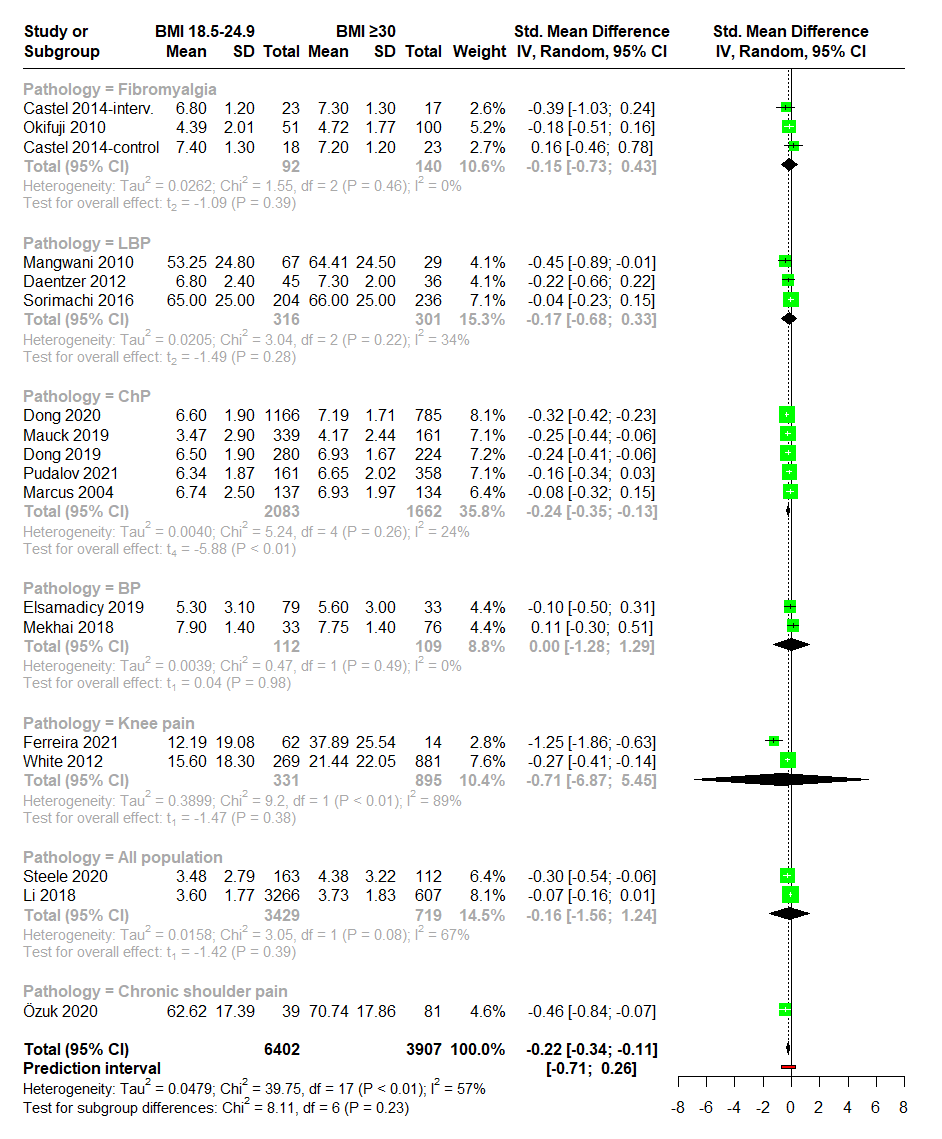


**Figure S5. Forest plot of subgroup analysis regarding pathology for studies assessing pain intensity in adults with normal weight (BMI = 18.5-24.9) *versus* adults with obesity (BMI ≥ 30).** Negative values indicate that pain intensities in adults of normal weight are lower than those for adults with obesity. SD: standard deviation; CI: confidence interval; LBP: low back pain; ChP: chronic pain; BP: back pain.


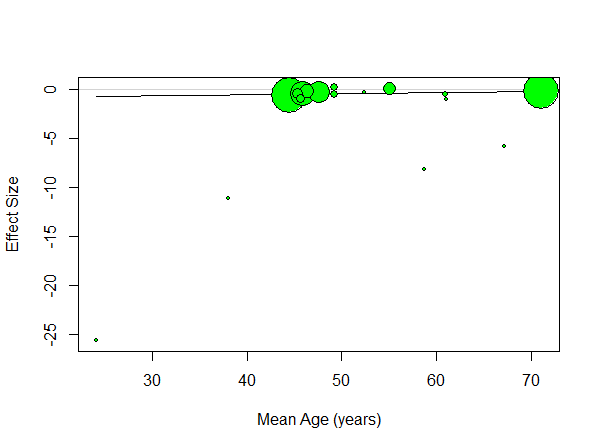


**Figure S6. Bubble plot (meta-regression) of age influence on between-group effect size for studies assessing pain intensity in adults with normal weight (BMI = 18.5-24.9) *versus* adults with obesity (BMI ≥ 30).**


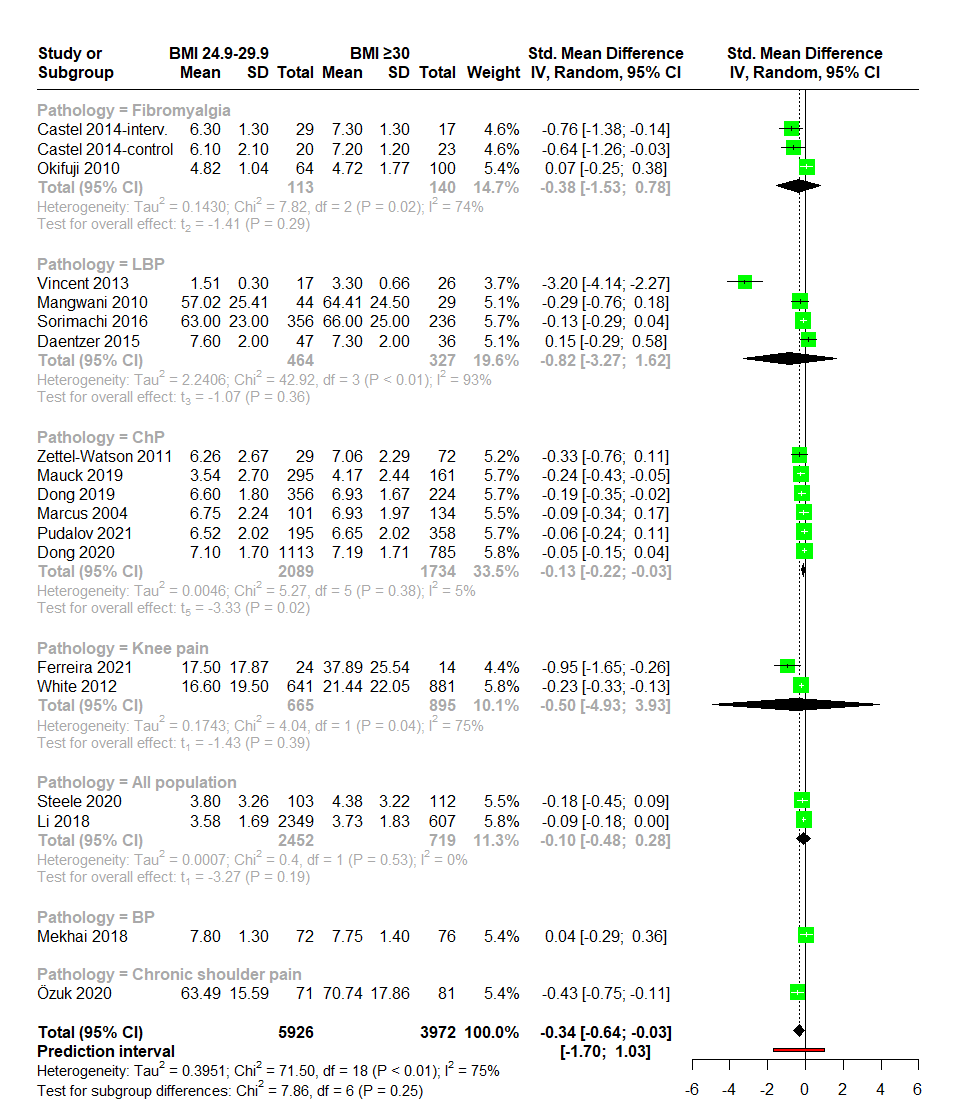


**Figure S7. Forest plot of subgroup analysis regarding pathology for studies assessing pain intensity in adults with overweight (BMI = 25-29.9) *versus* adults with obesity (BMI ≥ 30).** Negative values indicate that pain intensities in adults of overweight are lower than those for adults with obesity. SD: standard deviation; CI: confidence interval; LBP: low back pain; ChP: chronic pain; BP: back pain.


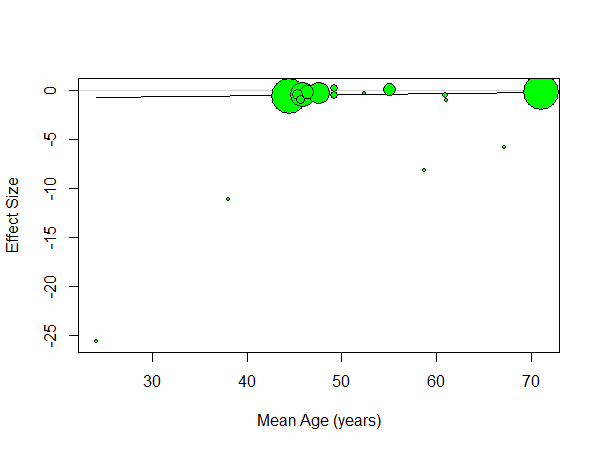


**Figure S8. Bubble plot (meta-regression) of age influence on between-group effect size for studies assessing pain intensity in adults with overweight (BMI = 25-29.9) *versus* adults with obesity (BMI ≥ 30).**


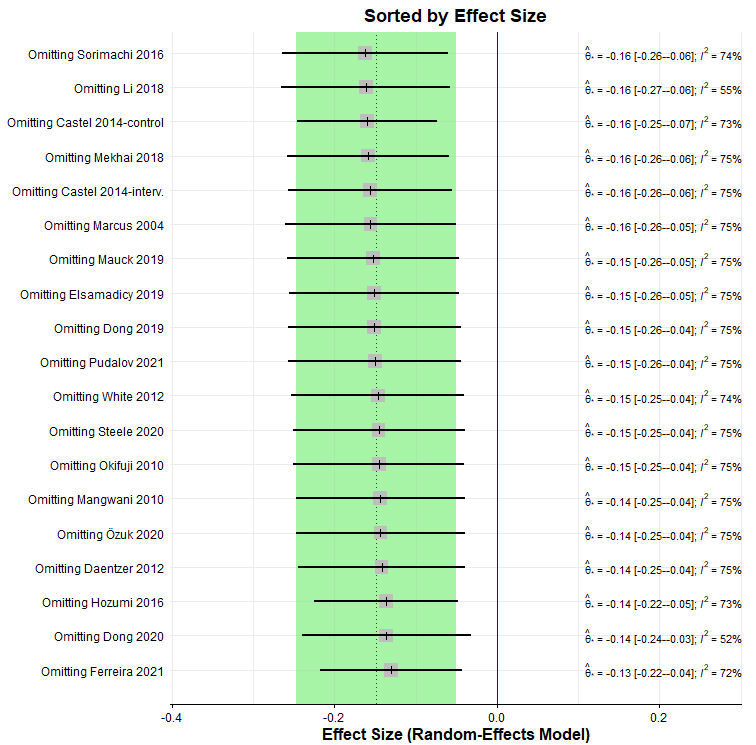


**Figure S9. Leave-one out analysis for the meta-analysis assessing pain intensity in adults with normal weight (BMI = 18.5-24.9) *versus* adults with excess weight (overweight and obesity) (BMI ≥ 25.0).** Negative values indicate that pain intensities in adults of normal weight are lower than those for adults with excess weight.


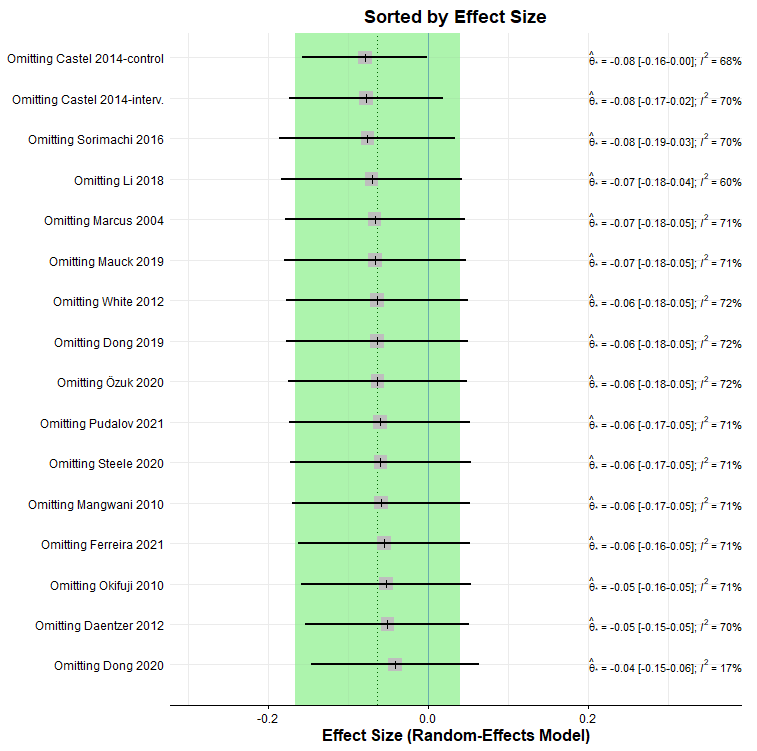


**Figure S10. Leave-one out analysis for the meta-analysis assessing pain intensity in adults with normal weight (BMI = 18.5-24.9) *versus* adults with overweight (BMI = 25-29.9).** Negative values indicate that pain intensities in adults of normal weight are lower than those for adults with overweight.


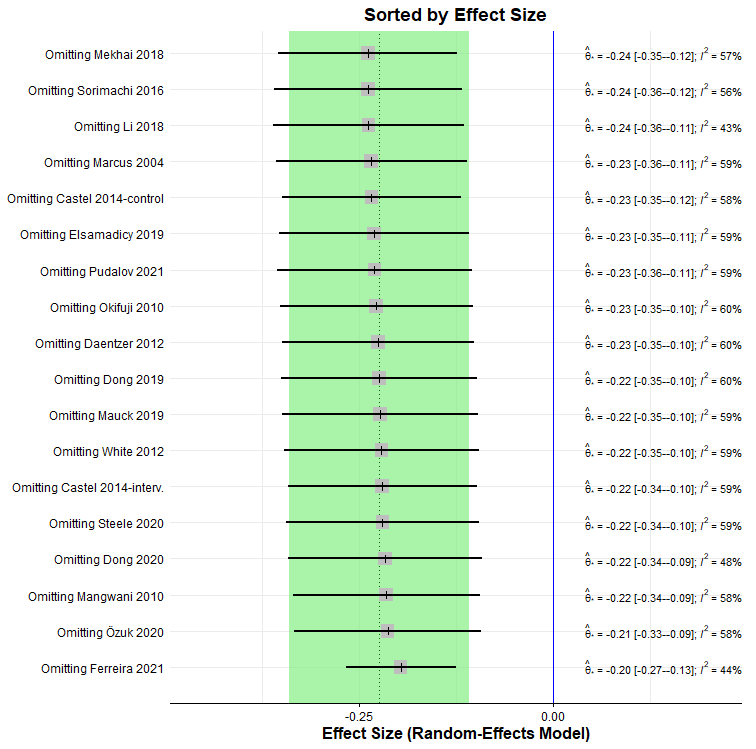


**Figure S11. Leave-one out analysis for the meta-analysis assessing pain intensity in adults with normal weight (BMI = 18.5-24.9) *versus* adults with obesity (BMI ≥ 30).** Negative values indicate that pain intensities in adults of normal weight are lower than those for adults with obesity.


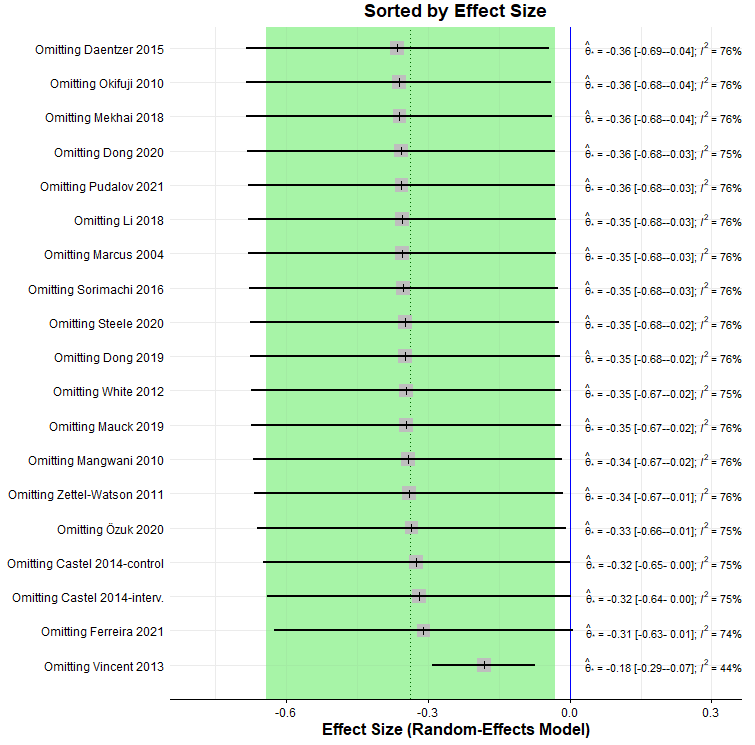


**Figure S12. Leave-one out analysis for the meta-analysis assessing pain intensity in adults with overweight (BMI = 25-29.9) *versus* adults with obesity (BMI ≥ 30).** Negative values indicate that pain intensities in adults of overweight are lower than those for adults with obesity.


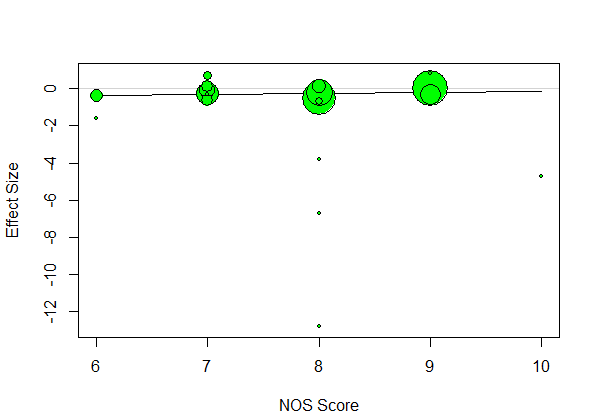


**Figure S13. Bubble plot (meta-regression) of NOS evaluation influence on between-group effect size for studies assessing pain intensity in adults with normal weight (BMI = 18.5-24.9) *versus* adults with excess weight (overweight and obesity) (BMI ≥ 25).**

**
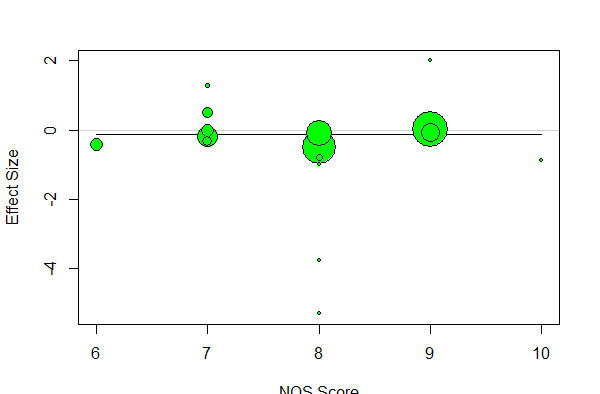
**

**Figure S14. Bubble plot (meta-regression) of NOS evaluation influence on between-group effect size for studies assessing pain intensity in adults with normal weight (BMI = 18.5-24.9) *versus* adults with overweight (BMI = 25.5-29.9).**

**
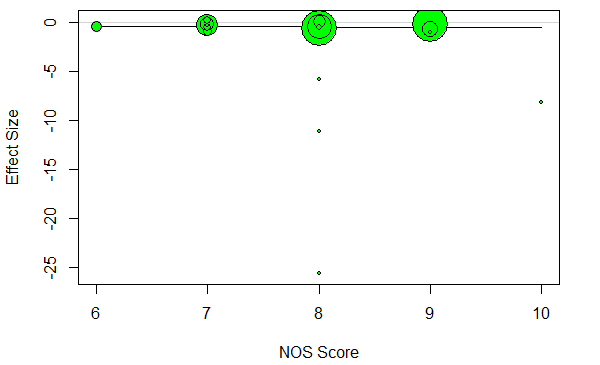
**

**Figure S15. Bubble plot (meta-regression) of NOS evaluation influence on between-group effect size for studies assessing pain intensity in adults with normal weight (BMI = 18.5-24.9) *versus* adults with obesity (BMI ≥ 30).**

**
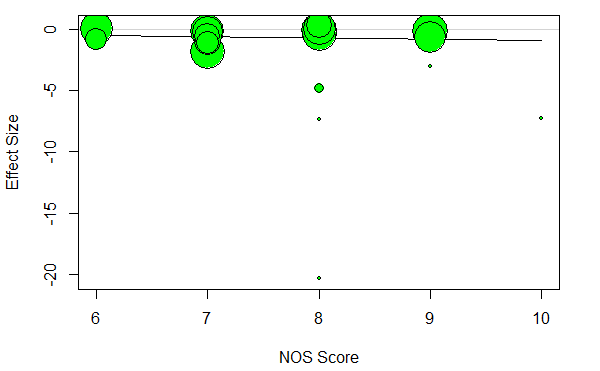
**

**Figure S16. Bubble plot (meta-regression) of NOS evaluation influence on between-group effect size for studies assessing pain intensity in adults with overweight (BMI = 25.5-29.9) *versus* adults with obesity (BMI ≥ 30).**


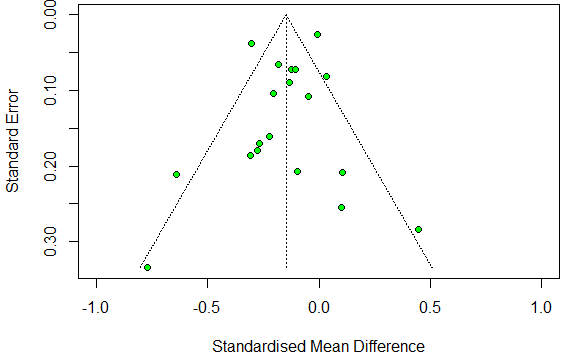


**Figure S17. Funnel plots for pain outcomes in studies comparing adults with normal weight (BMI = 18.5-24.9) *versus* adults with overweight and obesity (BMI ≥ 25).**

**
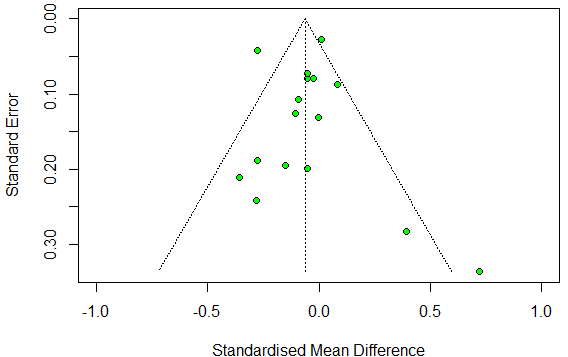
**

**Figure S18. Funnel plots for pain outcomes in studies comparing adults with normal weight (BMI = 18.5-24.9) *versus* adults with overweight (BMI = 25-29.9).**

**
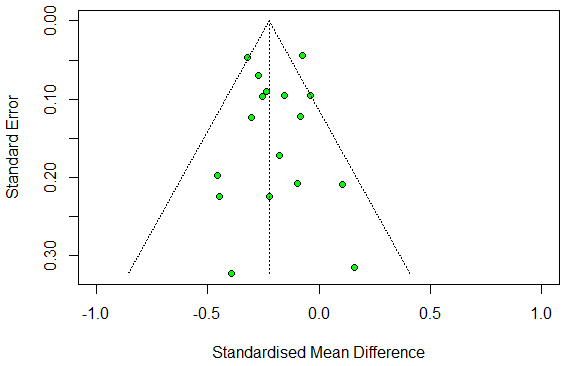
**

**Figure S19. Funnel plots for pain outcomes in studies comparing adults with normal weight (BMI = 18.5-24.9) *versus* adults with obesity (BMI ≥ 25).**

**
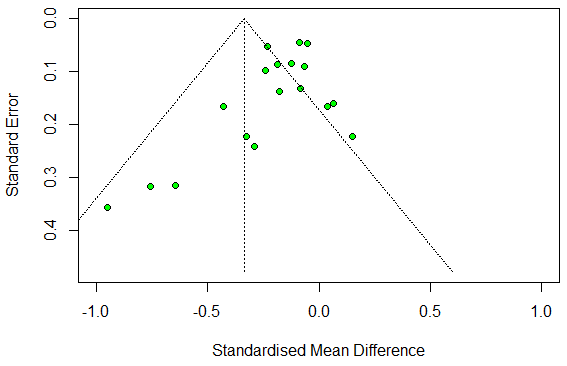
**

**Figure S20. Funnel plots for pain outcomes in studies comparing adults with overweight (BMI = 25-29.9) *versus* adults with obesity (BMI ≥ 25).**
